# Obesity, cancer risk, and time-restricted eating

**DOI:** 10.1007/s10555-022-10061-3

**Published:** 2022-08-19

**Authors:** Manasi Das, Nicholas J. G. Webster

**Affiliations:** 1 VA San Diego Healthcare System, San Diego, CA USA; 2 Department of Medicine, Division of Endocrinology and Metabolism, University of California, La Jolla, San Diego, CA USA; 3 Moores Cancer Center, University of California, La Jolla, San Diego, CA USA

**Keywords:** Obesity, Metabolism, Cancer, Dietary interventions time-restricted feeding

## Abstract

Obesity and the associated metabolic syndrome is considered a pandemic whose prevalence is steadily increasing in many countries worldwide. It is a complex, dynamic, and multifactorial disorder that presages the development of several metabolic, cardiovascular, and neurodegenerative diseases, and increases the risk of cancer. In patients with newly diagnosed cancer, obesity worsens prognosis, increasing the risk of recurrence and decreasing survival. The multiple negative effects of obesity on cancer outcomes are substantial, and of great clinical importance. Strategies for weight control have potential utility for both prevention efforts and enhancing cancer outcomes. Presently, time-restricted eating (TRE) is a popular dietary intervention that involves limiting the consumption of calories to a specific window of time without any proscribed caloric restriction or alteration in dietary composition. As such, TRE is a sustainable long-term behavioral modification, when compared to other dietary interventions, and has shown many health benefits in animals and humans. The preliminary data regarding the effects of time-restricted feeding on cancer development and growth in animal models are promising but studies in humans are lacking. Interestingly, several short-term randomized clinical trials of TRE have shown favorable effects to reduce cancer risk factors; however, long-term trials of TRE have yet to investigate reductions in cancer incidence or outcomes in the general population. Few studies have been conducted in cancer populations, but a number are underway to examine the effect of TRE on cancer biology and recurrence. Given the simplicity, feasibility, and favorable metabolic improvements elicited by TRE in obese men and women, TRE may be useful in obese cancer patients and cancer survivors; however, the clinical implementation of TRE in the cancer setting will require greater in-depth investigation.

## Introduction

Obesity has reached epidemic proportions globally, with nearly 39% of adults being classified as overweight and, of these, over 600 million being categorized as clinically obese in 2020 [1]. At the current pace, nearly half of the world’s population will be overweight or obese by 2030. Currently in the USA, 60% of the population is overweight and 30% is obese [2, 3]. The implications of this epidemic on the USA and global population health are enormous, as obesity has been linked to several metabolic, cardiovascular, and neurodegenerative diseases [4]. Furthermore, obesity is associated with an increased risk for developing cancer and predicts worse outcomes for a variety of malignancies [5–7]. Obesity may also worsen several aspects of cancer survivorship, including quality of life, cancer progression and recurrence, and disease-free survival [8]. Globally, 481,000 new cancer cases are attributed to overweight and obesity according to United Nations news report in 2014, establishing excessive body adiposity as a strong risk factor for cancer development [9]. The American Cancer Society reported in 2014 that 7.8% (122,536) of all cancers and 6.5% (38,188) of all cancer deaths in the USA were attributed to excess body weight [10]. After cigarette smoking, obesity represents the second greatest modifiable risk factor in the USA. The increased risk of cancer incidence and mortality is multi-factorial, but likely related to both the innate pro-inflammatory environment, dysregulation of growth factor and hormone expression, and altered circadian rhythms that occur in obesity. For instance, chronic low-level inflammation in viral hepatitis (a disease of the liver causing inflammation), obesity, or alcohol abuse is a risk factor for liver cancer [11]; increased levels of insulin and insulin-like growth factor-1 (IGF-1) may promote the development of colon, kidney, prostate, and endometrial cancers [12]; high levels of estrogen have been associated with increased risk of endometrial, breast, and ovarian cancer [13–15]; and circadian deregulation in night shift workers or in obesity has been connected with increased risk of breast cancer [10]. Given the common co-occurrence of obesity-related risk factors in many cancer patients that affect overall survival and increases risk of death, it is logical that strategies for weight control would be beneficial for both prevention and to improve cancer outcomes. Therefore, there is an urgent need to improve cancer care beyond novel therapeutics by elucidating the effects of different weight management strategies in cancer prevention and treatment. In this regard, many observational studies have provided consistent evidence that individuals with lower weight gain or weight loss have lower risk of colon cancer, kidney cancer, and breast, endometrial, and ovarian cancer [16–19]. Weight loss through dietary interventions such as caloric restriction (CR), intermittent fasting (IF), and fasting-mimicking diets (FMD) have beneficial metabolic effects and decrease cancer risk but are difficult to maintain. Surgical approaches such as gastric bypass are also beneficial in the short-term but long-term improvements are rare. Time-restricted eating (TRE) is a popular new intervention for improved metabolic health and weight control that does not involve calorie reduction. This method is a potentially easier way to maintain optimal body weight and health over a long period as it does not require reducing total food intake, calculating daily calorie intake, or changing diet. Small clinical studies have confirmed the effectiveness of this strategy to improve overall metabolic heath [20–22]. Preclinical studies have also reported the therapeutic benefits of TRE in mouse models of cancer [23–25]. Clinical trials are just starting to explore the role of TRE in cancer so it is too early to assess whether TRE has encouraging outcomes in cancer prevention and treatment. Although TRE is a promising dietary intervention for controlling weight and improving metabolic dysfunction in overweight or obese individuals, large-scale clinical trials are still needed to confirm the benefit of TRE for metabolic health and cancer prevention. In this review, we will give an overview of obesity as risk factor for cancer and the potentially useful role of time-restricted eating in cancer prevention and treatment.

## Obesity and cancer: overview of a complex relationship

Obesity is defined by a body-mass index (BMI) of > 30 and over-weight as a BMI of 25–29.9. These cutoffs have been developed based on Caucasian data and it is important to recognize that they may not hold for other groups. For example, the Asia–Pacific classification uses a BMI 23–24.9 for over-weight and > 25 as obese. Obesity has been associated with an increased incidence of a variety of cancers such as colorectal, kidney, esophagus, endometrium, breast, pancreas, thyroid, liver, ovary, gallbladder, and prostate cancer, as well as non-Hodgkins lymphoma [26, 27]. In addition, obesity is increasingly recognized as an indicator of poor prognosis as data show that obesity is associated with higher rates of cancer progression and recurrence, reduced progression-free survival, and increased mortality, especially for breast, prostate, and colon cancer [28–33]. Cancer metastasis accounts for over 90% of cancer mortality and obesity increases distal metastasis, thereby increasing the severity of the disease and mortality [34, 35]. Unfortunately, weight gain after diagnosis is common in cancer patients, especially among breast cancer patients receiving systemic adjuvant therapy [36, 37]. In a study of 535 women with newly diagnosed breast cancer, 84.1% of the patients gained weight during the first year after diagnosis and the weight gain was significantly greater in patients on chemotherapy [37]. Obesity also increases the risk of complications from cancer treatment and the risk of several comorbidities. For example, obesity is associated with an increased risk of both treatment-related lymphedema in breast cancer survivors [38] and incontinence in prostate cancer survivors who have undergone radical prostatectomy [39]. Thus, obesity represents a significant modifiable risk factor affecting cancer health worldwide.

The mechanisms underlying the cancer-promoting effect of obesity are complex and likely multifactorial. There are several potential explanations for the link between increased adiposity and worse cancer prognosis, including hormonal, inflammatory, and immune system effects. Studies have documented links between obesity and elevated levels of free circulating hormones (e.g., insulin and estradiol) and their impact on hormone-dependent cancers [15, 40–42] such as breast and prostate cancer. These differences likely underlie the reported differential effects of obesity on cancer subtypes. A large meta-analysis of breast cancer studies reported that obesity in premenopausal women is a positive risk factor for triple-negative breast cancers (TNBC, odds ratio (OR) 1.4–3.7) but a negative risk factor in estrogen receptor (ER) positive breast cancers (OR 0.35–0.81) [43]. In contrast, obesity in postmenopausal women is a positive risk factor for ER-positive breast cancer (OR 1.2–2.7) when endogenous estrogen levels are low. The detrimental effect of obesity is not limited to cancer risk however the American Cancer Society Prevention Study II of 495,977 women reported an association of BMI and BrCa mortality. Women with BMI > 30 kg/m^2^ had > 65% increase in mortality [5]. In the UK Prospective Study of Outcomes in Sporadic and Hereditary Breast Cancer (POSH) study of 2,956 young (aged < 41 yrs) breast cancer survivors, obesity was associated with larger tumors, positive lymph node status, and higher percentage of TNBC. Overall (8-year) survival and disease-free interval were significantly shorter [44]. Lastly, a meta-analysis of 82 studies including 213,075 breast cancer survivors found a 40% increased risk of mortality due to obesity in both pre- and post-menopausal women [45]. These observations might indicate that dietary interventions to reduce obesity may only be beneficial in selected cancer subtypes, but obesity has a detrimental effect on mortality in all subtypes of breast cancer, so one cannot be guided by cancer risk analyses alone.

Obesity has been linked to increases in estradiol due to aromatase expression in adipose tissue [46]. In the HEAL study of 505 postmenopausal women with stage 0-IIIA breast cancer, adiposity was positively associated with circulating levels of estrone and estradiol [47]. A combined meta-analysis of nine cohort studies, which included data from 663 breast cancer cases and 1,765 women without breast cancer, found that postmenopausal women with serum hormone concentrations in the top quintile for androstenedione, testosterone, dehydroepiandrosterone (DHEA), and DHEA-sulfate were nearly twice as likely to develop breast cancer in comparison to women with serum hormones in the bottom quintile [48]. In the same analysis, a doubling of androgen concentration resulted in a 20% to 40% increase in risk for breast cancer. Other hormones have also been implicated. One of the best documented effects of obesity is to cause hepatic insulin resistance that triggers a compensatory increase in insulin secretion to maintain normoglycemia. This results in fasting hyperinsulinemia. Other tissues including tumors do not become insulin resistant so are exposed to elevated insulin levels. Hence, increased signaling via insulin and IGF-1 receptors, and the downstream phosphatidylinositol 3-kinase pathway, are observed in diverse cancers [49]. For example, in non-diabetic breast cancer patients, higher levels of fasting insulin have been associated with a 2–threefold increased risk of mortality [50–54]. Similarly, the Women’s Health Initiative Observational Study (WHI-OS) of 93,676 postmenopausal women, insulin levels were associated with a > 2.4-fold increase in breast cancer risk in women not on hormone-replacement therapy [55]. The increased risk may be restricted to postmenopausal women as the Nurse’s Health Study II of 29,611 women did not show an association of insulin with breast cancer incidence [56]. Elevated insulin levels may also be associated with cancer progression. Additionally, fasting insulin levels were significantly associated with both distant recurrence and death. In a study, women in the highest quartile of insulin levels had a 2.1 times increased risk of distant recurrence compared to those in the lowest quartile (95% CI = 1.2–3.6, *P* = 0.01) and a 3.3 times greater risk of death (95% CI = 1.5–7.0, *P* = 0.002) [52]. Similar findings are reported for colorectal cancer [57]. A meta-analysis of all cancer deaths in non-diabetics reported that fasting serum insulin was associated with increased mortality (HR 1.92) in men [58] and the French TELECOM study reported that elevated fasting insulin posed increased risk of cancer death (HR 2.30) in men over a 28-year follow-up [59].

Chronic tissue inflammation is a feature of obesity. Inflammation in itself makes individuals susceptible to many forms of cancer as it has been linked to different steps involved in tumorigenesis, including transformation of normal cells to cancerous cells, survival, proliferation, promotion, invasion, angiogenesis, and metastasis [60]. Immune cells such as tumor-associated macrophages, tumor-associated dendritic cells, and pro-inflammatory cytokines and chemokines are key players in initiating inflammation creating a pro-cancer microenvironment [61]. Obesity is associated with inflammatory markers including C-reactive protein, serum amyloid A, interleukin-6, interleukin-1, and tumor necrosis factor alpha, and importantly some of these are higher in patients with metastatic cancer compared with patients without cancer and with those with early cancer [2].

### Circadian disruption in obesity and cancer

Circadian rhythms in physiology, metabolism, and behavior are vital part of homeostasis [62]. These rhythms occur from interactions between circadian clocks within brain and peripheral organs with cycles in light and dark, sleep and activity, and eating and fasting. Notably, obesity and its associated eating patterns have been shown to alter the circadian clocks in both the brain and peripheral tissues that generate 24 h rhythms in gene expression and diurnal behaviors [63–66]. Interestingly, daily rhythms in gene expression modulate several key aspects of cellular and tissue function with profound implications in disease prevention, and disease management including genes involved in glycolysis, gluconeogenesis, protein synthesis, lipid synthesis and oxidation, and mitochondrial function [67]. Acute circadian disruption can exacerbate chronic diseases, while chronic circadian disruption raises the risk for numerous diseases [62]. For example, forced circadian misalignment is associated with increased risk for obesity, diabetes, and cardiovascular disease. In a study involving ten adults (5 female) for 10-days, subjects were subjected to an artificial 28-h day, so they ate and slept at all phases of the circadian cycle during the 10-day stay. Subjects ate 4 isocaloric meals each 28-h day. When subjects ate and slept approximately 12 h out of phase from their normal 24-h circadian rhythms, increased both blood glucose and insulin (indicating insulin resistance), increased mean arterial pressure, reversed the daily cortisol rhythm, and reduced sleep efficiency. Notably, 3 of the 8 subjects developed a prediabetic state by this circadian misalignment [68].

Circadian clock disruption has been reported in some cancers and this is thought to promote tumor growth, owing to the dysregulation of key cell-cycle and tumor suppressor genes that are under clock control [69, 70]. In general, arrhythmic mice are susceptible to a variety of cancers [71–73]. In lung cancer, deletion of clock genes increases mutant Kras lung tumorigenesis [74]. Mechanistically, the loss of core clock gene components such as *Per2* and *Bmal1* leads to increased c-Myc expression, enhanced proliferation and metabolic dysregulation. A number of studies point to the role of MYC in both circadian disruption and cancer as it is a key player in cancer metabolism [75]. Deregulated expression of MYC or N-MYC disrupts the molecular clock by directly inducing REV-ERBα to dampen expression and oscillation of BMAL1, and both REV-ERBα and BMAL1 have key roles in N-MYC-driven human neuroblastomas. Importantly, these studies suggest a link between oncogenic transformation and circadian and metabolic dysrhythmia, which could be advantageous for cancer growth. In a similar study, overexpression of MYC in U2OS cells, severely attenuates circadian oscillations and promotes cell proliferation [76]. The authors showed that inhibition of the circadian clock was dependent on the formation of repressive complexes of MYC with MIZ1 leading to downregulation of the core clock genes *CLOCK*, *BMAL1* and *NPAS2*. Interestingly, cancer stem cells display robust circadian rhythm with exquisite dependency on core clock transcription factors, BMAL1 and CLOCK, for optimal cell growth. It has been demonstrated that knockdown of either *BMAL1* or *CLOCK* has been observed to induce cell cycle arrest and apoptosis in cancer stem cells in a patient-derived glioblastoma cell or murine leukemia stem cells in acute myeloid leukemia [77, 78]. Circadian disruption can also create a pro-tumor environment in the host. Chronic jet lag in mice induces persistent deregulation of liver gene expression and metabolism, promoting the development of spontaneous hepatocellular carcinoma [79]. Tumors may also influence normal circadian rhythms as Masri et al. demonstrated that lung cancer reprograms hepatic metabolism by rewiring hepatic circadian rhythms in gene expression and metabolites [80].

Epidemiological studies have also linked circadian disruption and clock genes to increased susceptibility to cancer development of diverse tissue types [for reviews see refs [81–86]. For example, there are several links between circadian clocks and breast cancer [71, 73, 87]. Women with SNPs in *CRY2, NPAS2*, and *CLOCK* are at a higher risk of breast cancer [88–90], and PER2 suppresses estrogen receptor-dependent transcription [73, 91, 92]. Low-grade and non-metastatic breast tumors have functional clocks, but aggressive carcinomas are arrhythmic [93]. Low CRY2 and PER1/2 expression is correlated with ER negativity, higher tumor grade and shorter overall survival in breast cancer patients [94, 95]. Breast cancer patients have higher methylation of the *CRY2* promoter consistent with lower *CRY2* expression [96] and loss of PER3 and CRY2 co-expression increases metastasis risk [93]. In hematological malignancies, *BMAL1* expression levels correlate inversely with *MYC* levels [76], the PER genes are downregulated in CLL [97], NPAS2 is up-regulated in AML patients [98], and the CRY genes show both up- and down-regulation in CLL and AML [99, 100]. Similar associations have been reported in other cancers, including head and neck [101], colorectal cancer [83, 102], liver cancer [103], and lung cancer [104, 105] to name but a few. Overall, the accumulated data point to the importance of circadian rhythms in normal health and suggest that interventions to normalize disrupted rhythms in obesity and cancer could be beneficial.

## Obesity management in cancer

Several methods for weight loss or control have been tested in the general population [106], including diets, exercise, and bariatric surgery [107–110]. Dietary interventions have received a lot of attention in both the scientific and lay community as a result of successful results in experimental animal models [111, 112]. The limited human data are consistent with the animal data. Sustained weight-loss after the age of 50 measured over 10 years reduces the risk of breast cancer (HR 0.68–0.82), whereas stable weight or short-term weight loss over one 5-year interval does not reduce risk [17]. This observation underscores the need for an intervention that is sustainable over a long period. Strong evidence for a causal link between obesity and cancer comes from bariatric surgery studies. Weight loss through bariatric surgery reduces the risk of colon, endometrial, pancreas, and pre-menopausal ER-negative and post-menopausal ER-positive breast cancer [113, 114]. Dieting or caloric restriction for weight loss can also prevent cancer. Experimentally, CR involves a 30% reduction in the daily caloric intake with the usual timing of meals [111] and CR without malnutrition remains the most robust intervention to date for cancer prevention in rodents, monkeys, and humans [111]. CR promotes anti-carcinogenic adaptations such as decreased production of inflammatory cytokines, growth factors, and anabolic hormones as well as decreased oxidative stress and DNA damage [115]. Despite of an abundance of the literature on the mechanisms and impact of CR, its clinical applicability remains limited because of challenges in long-term sustainability as most people regain weight lost during CR. Considering difficulties maintaining weight loss with CR, adopting a healthy diet to promote weight loss has been tested. A healthy diet, either with or without physical activity, however, does not alter disease-free survival or mortality in breast cancer [116]. Although physical activity does not alter cancer outcomes, there is evidence for a beneficial effect on quality-of-life, depression, anxiety, lymphedema, and fatigue [117].

## Health benefits of time-restricted eating

There has been growing interest in intermittent fasting as an alternative to CR because of promising results in experimental animal models [112]. According to a survey by the International Food Information Council Foundation, IF has become the most popular dietary intervention and many cancer patients are seeking advice from oncologists about its beneficial effect for cancer prevention and treatment [118]. IF can take various forms, including alternate day fasting with 0–25% of normal daily calories on the fasting days, the 5:2 method with 2 days of 25% calorie intake every 5 days of normal eating, periodic fasting (calorie intake is restricted for multiple consecutive days, such as 5 days, once a month, and unrestricted on all other days), Sunnah fasting (fasting every Monday and Thursday), and many other variations. Preclinical studies have shown beneficial effect of IF on tumor growth. In p53-deficient cancer mouse model, a 1 day per week IF regimen delayed tumor onset, significantly reduced tumor metastasis, and improved overall survival [119]. A study in a human xenograft prostate cancer model, an IF regimen comprised of 2 separate 24-h fasting periods per week exhibited similar trends toward delayed tumor growth and improved survival compared to an iso-caloric control group [120]. When combined with a fasting-mimicking diet, IF blocks TNBC and cancer stem cell escape in mice [121]. Interestingly, several short-term randomized clinical trials have indicated promising effects of alternate day fasting or a 5:2 diet in improving some cancer risk factors, including decreased fasting glucose, insulin, and leptin levels and increased adiponectin [22]. A small nonrandomized study of 23 women at increased risk for breast cancer found that IF for 2 days per weeks resulted in 4.8% reduction in body weight, an 8.0% reduction in body fat, and an improvement in insulin resistance over 4 to 5 weeks [122]. Similarly, IF of a ketogenic diet in patients with grade 2–4 astrocytoma decreased body mass and insulin levels [123]. IF for 24 h before and after chemotherapy reduced hematologic toxicity and promote recovery of chemotherapy-induced DNA damage [124]. IF also improved quality of life in cancer patients undergoing chemotherapy [125, 126].

**Fig. 1:**
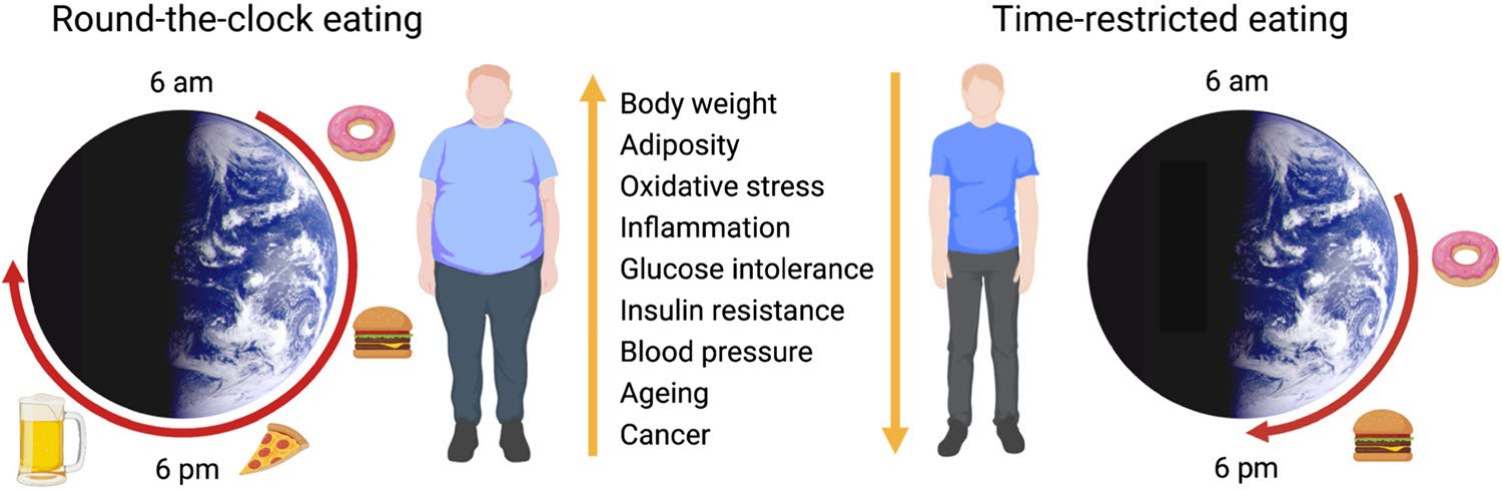
Health benefits of time-restricted eating

While IF emphasizes the ratio of fasting/feeding durations, time-restricted eating emphasizes the timing of eating within a limited window without involving CR. TRE is a type of intermittent fasting, which involves consuming all calories within a consistent 8–12 h daily window based on the normal circadian rhythm of eating (Fig. 1) [62, 67, 127]. TRE (also called time-restricted feeding or TRF in mice) improves metabolic health in animal models and potentially in humans and may facilitate adherence and long-term weight loss maintenance as it doesn’t involve calorie counting [22, 23, 128–130].

### Mouse Studies on Time-Restricted Eating

The metabolic benefits of TRE were first demonstrated in mouse model of diet-induced obesity [131]. Mice were given 8-h access to a high-fat diet (HFD) during the night (TRE), which is when mice are active, and compared to mice with 24 h access to food. The mice were protected against obesity, fatty liver, hyperinsulinemia, and inflammation and had enhanced motor coordination. Interestingly the mice on the TRE regimen consumed equivalent calories as those with ad libitum access. Furthermore, the TRE regimen improved mTOR, and AMPK signaling and enhanced circadian oscillations of core clock genes. These studies have been expanded to a variety of obesogenic diets and TRE at night prevented obesity and metabolic diseases without reducing caloric intake. The response showed a time-dependence with better effects with a 9-h feeding window compared to 12 or 15 h of feeding [132]. Interestingly, the protective effects were still maintained when TRE was interrupted by ad libitum access to food during weekends, a modified 5/2 regimen that is especially attractive for human lifestyle. Many studies, including ours, have demonstrated a similar beneficial effect of TRE in various mouse models to improve metabolic profiles [23–25, 133]. The metabolic improvement observed with TRE without any weight loss has led to the presumption that eliciting a daily fasting response, or at certain times of the day, is in itself beneficial. This would explain why dietary dilution, a form of CR in which mice eat all day to compensate for the low density of energy in their diet, does not result in lifespan extension. By the same argument, CR may improve health, at least in part, through an extended period of fasting. When considering TRE, it is important to recognize that meal-timing and circadian synchronization influences the metabolic effects. In a recent study, TRE extended the lifespan of Drosophila and was able to delay the onset of aging when flies were fasted during the night rather than during the day [134]. In mice, providing food during the first half of the active phase (earlyTRE) was more beneficial than providing during the second half (lateTRE) [135], and providing food during the day, rather than at night, disrupts liver circadian rhythms [136]. Timing of feeding may also extend the lifespan of mice on CR as Acosta-Rodriguez et al. demonstrated that CR with food provided for 12 h during the dark phase extended life span by 35% in C57BL/6 J mice whereas CR alone only extended the life span by 10% [137] and furthermore ameliorated the aging-associated changes in gene expression. In mice, TRE can impart benefits irrespective of nutrition quantity and quality and seems to be both preventive and therapeutic for aging and metabolic diseases [138].

### Human Studies on Time-Restricted Eating

Human data show a similar improvement in whole body metabolism (Table 1). For example, an isocaloric trial of TRE in pre-diabetic men for 5 weeks showed an improvement in glucose tolerance and a major decrease in systolic and diastolic blood pressure [20]. Another isocaloric study evaluating acute TRE for only 4 days showed a decrease in the average blood sugar level and reduced insulin resistance [139]. Likewise, a crossover-randomized trial [140] demonstrated that short-term TRE improved nocturnal glycemic control. Studies also support the impact of meal timing on metabolic health and indicate that eating at night is detrimental as it predisposes to obesity and metabolic dysregulation [141, 142]. For instance, women with metabolic syndrome on a daily three-meal schedule showed greater weight loss and metabolic improvement when the primary meal was at breakfast compared to women whose primary meal was at dinner [142]. In a small study with 19 men and women with metabolic syndrome, 10-h TRE reduced weight, blood pressure, and atherogenic lipids [130]. So, beneficial metabolic effects are seen in both sexes, which is consistent with studies in obese mice. Although, many metabolic studies support the beneficial effect of eating earlier in the day, not all studies support this idea. Evening protein ingestion leads to increased whole body and muscle protein synthesis [143], so TRE might not be advisable for sarcopenic patients. The effect of meal timing may even augment the impact of CR, as subjects in a weight-loss program who ate their main meal earlier in the day achieved greater weight loss than those subjects who ate later in the day [144], and in a separate study combined TRE and CR gave greater weight loss than CR alone although did not quite reach significance with the number of subjects studied [145]. Most human studies have focused on synchronizing the peripheral metabolic clocks to the central light-driven clock. It would be important to try TRE in individuals on night-shift workers with forced out-of-phase central and peripheral clocks or individuals with circadian rhythm sleep disorders [146] as mouse studies have shown desynchronization between central and peripheral clocks if food is provided during the daytime [147].

**Table 1:** A list of recent Time-Restricted Eating trials in humans and their key outcomes

Study Design	Duration	TRE Intervention	Participants	Age	Outcome	Study
Randomized control	12 weeks	TRE: 10 h, 8 am-6 pm	*n* = 60, diabetic	18–70 yr	↓ Body weight, HbA1c ↑ Insulin sensitivity	[148]
Randomized control	8 weeks	TRE: 10 h,	*n* = 60, obese	18–65 yr	↓ Body weight, Fasting glucose,	[149]
Randomized control	12 weeks	TRE: 8 h	*n* = 20 (17 females, 3 males), overweight	33–58 yr	↓ Body weight, lean mass, and visceral fat mass	[22]
Longitudinal	12 weeks	TRE: 8 h (10 am–6 pm)	*n* = 14, overweight	25–65 yr	↓ Body weight, fat mass, systolic blood pressure ↔ Gut microbiome	[150]
Cross-over	5 days	TRE: 8 h (10 am–6 pm) Extended eating: 15 h (7 am–10 pm)	*n* = 11 males, overweight	32–43 yr	↓ Night-time glucose, glucose and insulin iAUC after lunch ↔ Daytime glucose ↑ TG after lunch	[140]
Longitudinal	12 weeks	TRE: 10 h (self-selected, dinner before 8 pm): Baseline: ≥ 14 h	*n* = 19 (6 females, 13 males), overweight	48–70 yr	↓ Body weight, fat mass, waist circumference, blood pressure, plasma cholesterol ↔ Fasting glucose, HbA1c, HOMA-IR, fasting insulin	[130]
Longitudinal	4 weeks	TRE: 8 h	*n* = 10 (6 females, 4 males), overweight,	≥ 65 yr	↓ Body weight ↑ Quality of life	[151]
Longitudinal	13 weeks	TRE: 8–9 h	*n* = 40 (31 females, 9 males), with abdominal obesity	36–62 yr	↓ Waist circumference, HbA1c	[152]
Randomized control	8 weeks	TRE: 8 h (12 pm–8 pm) TRE plus β-hydroxy β-methyl butyrate	*n* = 40 females, resistance trained normal weight	18–30 yr	↓ Fat mass ↑ Muscle performance	[153]
Cross-over	4 days	TRE: 6 h (8 am–2 pm)	*n* = 11 (4 females and 7 males), overweight	25–39 yr	↓ Mean 24-h glucose, glycemic excursions, morning ghrelin, desire to eat ↑ metabolic flexibility, fullness, plasma ketones, fat oxidation	[139, 154]
Cross-over	1 week	Early TRE: 9 h (8 am–5 pm) delayed TRE: 9 h (12 pm–9 pm)	*n* = 15 males, overweight	52–58 yr	↓ Body weight, fasting TG, and hunger ↓ Mean fasting glucose by CGM in eTRE ↑ Glucose tolerance	[21]
Cross-over	5 weeks	TRE: 6 h (8 am–2 pm, dinner before 3 pm)	*n* = 8 males, overweight	47–65 yr	↓ Fasting TG, desire to eat in the evening ↑ Insulin sensitivity, β cell responsiveness ↔ Body weight	[20]
Historical control	12 weeks	TRE: 8 h (10 am–6 pm)	*n* = 23 (20 females, 3 males), obese	25–65 yr	↓ Body weight and blood pressure ↔ Fat mass, fasting glucose, LDL cholesterol, TG	[155]
Randomized control	8 weeks	TRE: 4 h (anytime 4 pm to midnight) for 4 days a week	*n* = 18 resistance trained males normal weight	18–27 yr	↔ Body weight, fat mass	[156]
Randomized control	8 weeks	TRE: 8 h (1 pm–8 pm)	*n* = 34 males, normal weight	25–33 yr	↓ Fat mass, fasting glucose, fasting insulin, total testosterone, IGF-1, inflammation	[157, 158]
Longitudinal	16 weeks	TRE: 10–11 h (self-selected)	*n* = 8 (3 females, 5 males), overweight	> 18 yr	↓ Body weight Improved sleeping	[159]
Cross-over	7 days	eTRE 70% calories before 5 pm vs TRE 8 h window vs ADF	*n* = 32 (25 females, 8 males), obese	mean age 45.7 yr	No difference in weight loss between diets. TRE easiest to follow	[160]
Longitudinal	12 weeks	eTRE + 35%CR 10 h (self-selected) vs 35% CR alone	*n* = 81 (69 females, 12 males	mean age 38 yr	No difference in weight loss	[161]
Longitudinal	12-weeks	TRE 8 h (self-selected) + CGM vs Control group 12 h	*n* = 50 (14 males, 36 females), obese	14–18 yr	No difference in weight loss	[162]
Longitudinal	5-weeks	eTRE 8 h (6am-3 pm) vs mTRE 8 h (11am-8 pm) vs Control	*n* = 82 (64 females, 18 males), normal weight	mean age 31	Weight loss and improved HOMA-IR in eTRE group	[163]
Longitudinal	10-weeks	TRE 10 h (8am-6 pm)	*n* = 15 (males), overweight	40–70 yr	↓ Body weight Improved GTT, ↓ Fasting glucose, HbA1c	[164]
Longitudinal	8-weeks	TRE 8 h (10am-6 pm or 12 pm-8 pm) vs Control	*n* = 30 (females), normal weight	40–65 yr	↓ Body weight ↓ Diastolic BP	[165]
Cross-over	3-days	early dinner (6 pm) vs late dinner (9 pm)	*n* = 12 (2 males, 10 females)	> 20 yr	↓ Mean 24 h glucose ↓ RQ after breakfast	[166]
Cross-over	4-weeks	TRE 8 h (1 pm-9 pm)	*n* = 12 (males), healthy	mean age 22 yr	↑ Exercise performance ↑ Fat-free mass	[167]
Longitudinal	3-months	TRE 10 h (10am-7 pm)	*n* = 50 (41 females, 9 males), overweight	30–75 yr	↓ Body weight ↓ Systolic BP	[168]
Longitudinal	8-weeks	TRE 8 h (12 pm-8 pm) + Exercise vs Exercise alone	*n* = 21 (18 females, 3 males), overweight	35–60 yr	↓ Body weight ↓ Fat mass	[169]
Longitudinal	12-weeks	TRE 8 h	*n* = 20 (17 females, 3 males), overweight	mean 45 yr	↑ Quality of life	[170]
Longitudinal	6-weeks	TRE 8 h (8am-4 pm)	*n* = 18 women with PCOS	18–31 yr	↓ Body weight ↓ Fat mass ↓ Fasting insulin ↓ HOMA-IR	[171]
Longitudinal	12-weeks	TRE 8 h	*n* = 20 (17 females, 3 males), overweight and obese	18–65 yr	↑ Bone mineral content	[172]
Longitudinal	6-months	TRE 12 h (self-selected) vs standard dietary advice	*n* = 213 (152 females, 61 males), normal to overweight	> 18 yr	↓ Body weight in TRE group	[173]
Longitudinal	10-weeks	TRE 4 h (3-7 pm) vs TRE 6 h (1-7 pm) vs Control	*n* = 58 (53 females, 5 males), obese	> 18 yr	↓ Body weight and insulin resistance in TRE groups, no diff 4 h vs 6 h	[174]
Longitudinal	12-weeks	TRE 8 h (self-selected)	*n* = 51 (37 females, 14 males), obese	> 18 yr	↓ Body weight in TRE group	[175]

↓ reduced; ↑increased; ↔ no change; iAUC, incremental area under the curve; BP, blood pressure; PCOS, poly-cystic ovary syndrome

## Time-restricted eating and cancer

Given that TRE improves metabolic health in obese animals and humans, it might be expected to have anti-cancer effects in obesity-driven cancers. This has been borne out in a few rodent studies that evaluated the effect of TRE in modulating cancer risk or progression. In a recent study using mouse postmenopausal breast cancer models, our group reported that TRE, in the absence of caloric restriction or weight loss, could effectively inhibit the accelerated tumor initiation, progression, and metastasis due to obesity in comparison with mice with 24-h access to food. This beneficial effect of TRE was mediated, in part, by reduced insulin signaling as systemic insulin infusion through implanted pumps reversed the TRE-mediated protection and reducing insulin secretion mimicked the protection [23]. Sundaram and Yan have also shown that TRE of high-fat diet prevented cancer in the same transgenic MMTV-PyMT model of spontaneous breast cancer [24]. This group also demonstrated that TRE prevented high-fat diet enhanced metastasis in a subcutaneously injected Lewis lung cancer mouse model [25]. Aging increases the risk of cancer, and it has been proposed that the aged tissue microenvironment provides a pro-neoplastic niche. A recent study demonstrated that TRE could prevent the aging-associated changes in microenvironment and consequently decreases the growth of transplanted pre-neoplastic hepatocytes [176]. Colorectal cancer is also sensitive to the intestinal microenvironment and dysregulation of the gut microbiome has been connected to the pathogenesis of colorectal cancer. TRE was recently shown to improve the gut microbiota and prevent colon cancer [177]. Not all cancers respond to TRE however. Turbitt et. al. tested whether TRF alone or combined with anti-CTLA-4 immunotherapy would reduce tumor growth a murine model of kidney cancer. They found that TRF alone did not reduce tumor growth or metastasis in lean chow-fed or obese HFD-fed mice. Immune-checkpoint therapy had no effect in chow-fed mice but did reduce tumor growth in normal weight and obese mice on HFD irrespective of TRF [178]. Similarly, mice harboring LAPC-4 prostate cancer tumors did not show decreased tumor growth or increased survival [179].

As large prevention studies are lacking, most human studies to date have been epidemiological studies or small studies focused on assessing cancer biomarkers. In the Women’s Healthy Eating and Living study on a cohort of 2413 women with breast cancer, there was a significant increase in the risk of breast cancer recurrence with fasting < 13 h per night compared to fasting > 13 h per night (hazard ratio, 1.36; 95% CI, 1.05–1.76) [180]. An analysis of the NHANES data showed that each 3-h increase in night-time fasting was associated with improved glucose regulation and a decrease in hemoglobin A1c [181]. A case–control study in 922 Chinese women with incident BrCa and 913 controls [182] reported that eating after 10 pm was significantly associated with increased risk of breast cancer (OR 1.50). The association was strongest in women who had > 20 year history of eating after 10 pm (OR 2.28). A population case–control study of 1205 breast cancers and 621 prostate cancers in 1321 women and 872 men in Spain reported that a longer interval between the last meal and sleep was associated with lower cancer risk (prostate OR 0.74, breast OR 0.64)[183]. Similar protection was reported if meal eaten before 9 pm vs after 10 pm (OR 0.75 & 0.85) and in morning chronotypes (OR 0.65 & 0.67). As mentioned earlier, obesity causes hyperinsulinemia that can drive tumor growth and reducing insulin levels in mouse models inhibits tumor growth. Indeed, most of the obesity-associated increased risk for breast cancer can be accounted for by the increased risk due to the hyperinsulinemia [184–186]. Several small TRE studies have reported reductions in insulin resistance, and by inference insulin levels, that would be expected to reduce cancer risk [20, 187]. Breast cancer risk is also linked with hypertension, with several studies reporting a 7–38% higher risk of breast cancer among women with hypertension [188]. A meta-analysis of six TRE studies with 97 participants showed clinically significant decreases in systolic and diastolic blood pressure [128, 189]. All these epidemiological and observational studies support the potential beneficial role of TRE in cancer. Nonetheless, these findings strongly suggest that more TRE studies are needed to better understand the underlying mechanisms and differences in outcomes before clinicians may start to consider safely and confidently prescribing TRE for the treatment of cancer in humans.

## What are the mechanisms underlying the beneficial effect of time-restricted eating?

As discussed earlier, obesity is tightly linked to the metabolic syndrome which is a collection of metabolic disturbances including hyperglycemia, hyperinsulinemia, dyslipidemia, and hypertension, many of which have been linked to cancer [190]. In a recent review, Mattson et al. discussed the metabolic and physiological responses to CR, IF, and TRE, and highlighted the importance of four mechanisms including the adaptive stress response to oxidative damage, the bioenergetics or normal and cancer cells, suppression of inflammation, and induction of autophagy to remove or repair damaged organelles [191]. Many of these pathways also have relevance to cancer development. Post-prandial hyperglycemia may provide excess glucose to cancer cells to support their rapid growth since many cancer cells are more glycolytic than normal cells [12, 192]. Hyperglycemia can cause overproduction of advanced glycation end-products and reactive oxygen species, which can cause DNA damage and may initiate cancer. Obesity can also cause oxidative stress through increased mitochondrial oxidation of lipids [193–196] and preliminary evidence suggests that TRE may reduce oxidative stress in men [20]. At the metabolic level, hyperinsulinemia increases the risk of both cancer incidence and death [197, 198]. This increase of cancer mortality is also observed in non-obese people with hyperinsulinemia [199]. Indeed, we recently demonstrated that TRF acts by correcting insulin resistance to prevent and inhibit breast tumor growth in mouse models of breast cancer [23]. Furthermore, obesity and diabetes alters the production of endotrophin, leptin, adiponectin, angiopoietins, bone morphogenic proteins, and other adipokines, which can also affect cancer cell growth and survival [200–204]. For example, endotrophin, which is a carboxy-terminal proteolytic cleavage product of collagen 6α3, is overexpressed in obesity, enhances progression of breast and liver cancer, enhances epithelial-mesenchymal transition, and causes chemoresistance [205–207]. As discussed earlier, obesity creates a state of sub-clinical, chronic tissue inflammation with immune cell infiltration due to elevated adipocyte inflammatory cytokine production [208, 209]. Such local inflammatory changes in the microenvironment have been shown to accelerate tumor initiation and growth [60, 61]. TRE reduces tissue macrophage infiltration and inflammation in mouse models [23, 131, 135, 210, 211]. Some human studies have shown that restricting food intake to 8 h, or a longer nighttime fast, significantly decreases proinflammatory markers [157] but other studies have not seen any changes in these markers [174, 212, 213].

In addition to the above-mentioned metabolic/inflammatory mechanisms, another mechanism to consider is circadian realignment. Most time-restricted eating protocols involve limiting food intake to a prescribed window, usually 6–10 h, but the timing of this window is also important. TRE during the normal active phase is more beneficial than TRE during the inactive phase in both animal and human studies. In-phase TRE reinforces the normal circadian rhythms of nutrient dependent clock genes, but out-of-phase TRE causes a phase shift in the normal oscillations. The circadian clock is essential for normal metabolic regulation and disruption of the clock causes obesity and insulin resistance [214–217]. Disruption of the clock also causes abnormal cellular division and promotes tumorigenesis [62, 69]. Indeed, clock genes have been implicated in cancer as many tumors are acyclic with deficient endogenous clocks [93, 218, 219], circadian gene variants are associated with cancer [89, 220], clock genes regulate oncogene expression and suppresses oncogenic signaling [221–223], and oncogenes regulate clock gene expression [224]. Our group has demonstrated that many of the disrupted tumor circadian rhythms were restored by TRE to patterns found in the normal tissues suggesting that TRE might suppress tumorigenesis by regulating tumor clock genes [23]. Despite the strong connection between circadian clock genes and cancer, no studies have shown a causative link between TRE-induced clock gene rhythms and tumor inhibition.

## Time-restricted eating safety

Fasting has been safely practiced by individuals in various religious practices. For instance, over the 30 days of Ramadan, individuals fast from dawn-to-dusk which varies up to 21 h per day depending on latitude, and in Judaism individuals routinely undertake 25 h fasts [212, 225–227]. TRE is distinct from these religious fasts as the long fasting period is overnight rather than during the day, so is less associated with hunger. TRE also does not require total withdrawal from food and drink, as water and other zero-calorie beverages are allowed. Importantly, TRE has been reported not to cause major adverse events or negatively impact eating disorder symptoms among adults with obesity, metabolic syndrome, diabetes [128, 140], or pre-diabetes [20, 129], and TRE with a daytime feeding time window of 8 h does not cause occurrences of hypoglycemia, nor cause depression, anxiety or stress [228]. TRE has proven to be a more effective, safe, and convenient strategy than CR diet to lose weight [229, 230]. In obese individuals, TRE preserves healthy muscle in contrast to CR that causes 20–35% muscle loss [231–234]. This is an important finding, because weight loss interventions typically result in concomitant decreases in both fat and lean body mass [156, 157]. However, safety studies of longer duration are needed before recommending TRE as a healthy lifestyle intervention for body weight control. Furthermore, TRE may not be suitable for everyone, especially those with underlying metabolic conditions. Adhering to a TRE diet is likely not wise for type 1 diabetics, since metabolic switching, which can occur with TRE, may lead to diabetic ketoacidosis [235]. Similarly, the potential use of TRE in pediatric intensive care units may be complicated by the susceptibility of newborns and infants to fasting-induced ketogenesis [236]. People with impaired liver function may also be particularly sensitive to TRE [237, 238].

## Time-restricted eating feasibility and adherence

TRE is a new treatment strategy for weight control, metabolic improvement, and diverse disease prevention without calorie reduction [190]. This method is an easier approach to maintain optimal body weight and health for a longer time because patients do not need to reduce total food intake, or calculate total daily calorie intake, or change the composition of their diet. Clinical studies have confirmed the effectiveness of this strategy. Dorothea et al. have reported that, 86% of participants achieved their weight target during the 3-month study period and TRE was well accepted by participants [152]. Studies in humans and animal models have reported the beneficial effects of TRE on obesity, diabetes, fatty liver, cardiometabolic dysfunctions, and lifespan [155, 239]. Several key features of TRE promote adherence relative to CR or other forms of IF. As TRE follows a cycle of fasting during the night with an 8–10 h eating window during the day with no calorie restriction, it may require less cognitive effort and facilitate dietary satisfaction. Additionally, TRE may reduce conflict with the homeostatic drive to eat and prevent dietary lapses resulting from prolonged negative energy balance [240]. In a large, randomized controlled trial of TRF in 116 overweight and/or obese men and women, high adherence to the TRF protocol (8-h feeding window) was reported [241]. Follow-up data from two small TRE trials reported promising data that subjects continued TRE even after the trial period had ended. In one study, long-term follow-up ~ 16 months after the end of the study reported that > 60% of the participants were still practicing some form of TRE [130]. In another study, it was reported that all participants were still doing TRE and maintained their weight loss one year after the end of the study [159]. While these observations are anecdotal, they do support the idea that TRE is easy to adopt and maintain. Long term adherence is very important if TRE is to have any preventative value for cancer, as Teras et al. found that sustained weight loss over two successive 5-year periods was needed to show a decreased risk of breast cancer, weight loss of a single 5-year period did not show a protective effect [17]. Although TRE may be easy to maintain once adopted, there are potential barriers to trying TRE in the general population. Work and family schedules may make adherence to a strict eating window difficult. Luckily, the animal data has shown that the benefits of TRE are maintained even if performed only during the week. Human data are lacking, but if this finding holds true, a five day "weekends-off" TRE regimen may prove attractive allowing participation in social events while maintaining adherence to TRE [242].

## Conclusion and future directions

In conclusion, TRE is a promising therapeutic strategy for controlling weight and improving metabolic dysfunctions in those who are overweight or obese. As obesity represents a potential risk factor in cancer development and outcome, strategies that effectively modify obesity could potentially be harnessed as a means of cancer control. Preclinical studies support the potential beneficial effect of TRE in cancer prevention and growth. While definitive clinical trials showing the long-term effect of TRE on cancer prevention, treatment, and outcome are under investigation (Table 2), short-term TRE strategies for weight control may be helpful for some cancer patients and survivors. On a note of caution, TRE should still be regarded as a new dietary intervention with limited studies that have given mixed outcomes. For instance, small TRE studies have found significant decreases in weight and associated metabolic parameters, however, a large, randomized controlled trial of TRE in 116 overweight/ obese men and women for 12 weeks did not show a significant change in weight compared with the control group, although there were no measurements of energy intake or expenditure [241]. Therefore, large randomized clinical trials showing efficacy of TRE in obese individuals for 5-years or longer are needed before the adoption of TRE in the cancer clinical setting.

**Table 2:** List of ongoing clinical trials on TRE and cancer

Study	ClinicalTrials.gov Identifier:	Status	Disease condition, *n*	Time frame	Summary
Time-restricted Eating in Cancer Survivorship: A Single-arm Feasibility Pilot Study	NCT04243512	Active, not recruiting	Cancer survivor, *n* = 40	10 h TRE, 14 days	The investigators will assess the feasibility of delivering a time-restricted eating (TRE) intervention among cancer survivors with fatigue
Time-Restricted Eating (TRE) Among Endometrial Cancer Patients (TREND)	NCT04783467	Recruiting	Endometrial cancer patients, *n* = 15	8–10 h TRE, 16 weeks	The long-term goal of this study is to determine the efficacy of Time-Restricted Eating (TRE) for improving metabolic health, preventing cardiometabolic comorbidities, and improving prognosis after endometrial cancer diagnosis. The study will also evaluate the feasibility, fidelity and preliminary acceptability of TRE among endometrial cancer patients
Time-Restricted Eating and Cancer: Clinical Outcomes, Mechanisms, and Moderators	NCT04722341	Recruiting	Colorectal cancer, *n* = 300	8 h TRE starting 1–3 h after waking up, 6 months	The purpose of this study is to test whether the timing of meals can improve treatment adverse events, influence tumor biology and alter a person’s mood and behaviors
Time-Restricted Eating During Chemotherapy for Breast Cancer	NCT05259410	Recruiting	Breast Cancer, *n* = 40	8 h TRE staring 10am-6 pm, 12 weeks	The study will demonstrate that time-restricted eating, a form of intermittent fasting, will improve treatment related outcomes, patient related outcomes, and limit treatment related weight gain and fat mass accretion
Time-Restricted Eating (TRE) Among Native Hawaiian/Pacific Islander Women at Risk for Endometrial Cancer (TIMESPAN)	NCT04763902	Recruiting	Endometrial Neoplasms *n* = 30	8–10 h TRE, 14 weeks	The primary objective of the study is to evaluate the feasibility, fidelity and preliminary acceptability of a TRE intervention among Native Hawaiian/Pacific Islander women at risk for developing endometrial cancer and to provide proof of principle that TRE can improve metabolic health in this population
Time-restricted Eating Versus Daily Continuous Calorie Restriction on Body Weight and Colorectal Cancer Risk Markers	NCT05114798	Not yet recruiting	Colorectal Cancer *n* = 255	8 h TRE starting from 11am – 7 pm, 1 month	This research will demonstrate that time-restricted eating, a type of intermittent fasting, is an effective therapy to help obese individuals reduce and control their body weight and prevent the development of colorectal cancer
Time-Restricted Eating to Address Persistent Cancer-Related Fatigue	NCT05256888	Not yet recruiting	Cancer Survivor, *n* = 30	10 h TRE, 12 weeks	This study will assess the feasibility of delivering a 12-week time-restricted eating intervention as well as the intervention’s preliminary efficacy on persistent cancer related fatigue among cancer survivors
Metformin and Nightly Fasting in Women With Early Breast Cancer	NCT05023967	Not yet recruiting	Breast Cancer, *n* = 120	8 h TRE, 4–6 weeks (until surgery)	This study will explore the combined effect of prolonged nightly fasting and metformin hydrochloride extended release in decreasing breast tumor cell proliferation and other biomarkers of breast cancer
Effects of Time-Restricted Feeding on AGE-RAGE Signaling	NCT05038137	Not yet recruiting	Pre Diabetes Breast Cancer, *n* = 48	8 h TRE, 3½ months	This study will explore the TRE on metabolic changes in women at high risk of breast cancer
Impact of Metabolic Health Patterns And Breast Cancer Over Time in Women	NCT05432856	Recruiting	Breast Cancer, *n* = 65	8 h TRE, 24-weeks	This study will examine changes in fat volume, liver fat, metabolic syndrome score, Framingham risk score, peak VO2, insulin resistance, changes in hormonal markers and cytokines
Intermittent Fasting Accompanying Chemotherapy in Gynecological Cancers	NCT03162289	Recruiting	Breast or Ovarian Cancer, *n* = 150	10 h TRE with 60–72 h modified fast during chemotherapy	Primary outcome will be FACT-G score, with complete remission or Millar Payne classification as secondary outcomes
Proof-of-Concept of Time-Restricted Eating as a Novel Lifestyle Intervention for Breast Cancer Prevention	NCT05454943	Recruiting	Women over 50 with metabolic dysfunction, *n* = 178	8 h TRE standard or personalized, with peer or external support	This study will assess adherence and HbA1c, with HOMA-IR, glucose control, body weight, metabolic syndrome score as secondary outcomes
Effect of Prolonged Nightly Fasting on Immunotherapy Outcomes in HNSCC—Role of Gut Microbiome	NCT05083416	Recruiting	Adults with newly diagnosed recurrent/metastatic HNSCC, *n* = 52	8–10 h TRE, 3-months	Primary outcome will be adherence, with changes in gut microbiome as secondary outcome
Intermittent Fasting and CLL/SLL	NCT04626843	Active, not recruiting	Adults with CLL or SLL, *n* = 15	8 h TRE, 3-months	This study will assess change in lymphocyte count, quality of, life, inflammation, metabolic profile, autophagy and immune cell gene expression
Metabolic Therapy Program In Conjunction With Standard Treatment For Glioblastoma Multiforme	NCT04730869	Recruiting	Newly diagnosed GBM, *n* = 22	2 × 1 h eating intervals with ketogenic diet between 2 5-day fasts during chemotherapy	Primary outcome will be glucose-to-ketone ratio, with changes in weight, quality of life, activity, adverse events, progression-free and overall survival as secondary outcomes
